# An Optimized Microwave-Assisted Extraction and Evaluation of Amino Acids Content and Nutritional Value in *Chebulae fructus* from Different Origins

**DOI:** 10.3390/foods14071166

**Published:** 2025-03-27

**Authors:** Zhiqi Xu, Yan Li, Yuan Shen, Yiwu Wang, Jialing Yu, Xinxin Xiang, Lin Yang, Dan He

**Affiliations:** 1 College of Pharmacy, Chongqing Medical University, Chongqing 400016, China; xuzhiqi886@163.com (Z.X.); sybonnie@cqmu.edu.cn (Y.S.); willwyw@cqmu.edu.cn (Y.W.); 19923654482@163.com (J.Y.); 19122157313a@sina.com (X.X.); 2 Chongqing Key Laboratory of High Active Traditional Chinese Drug Delivery System, Chongqing Medical and Pharmaceutical College, Chongqing 401331, China; ly20031079@163.com

**Keywords:** *Chebulae fructus*, microwave-assisted extraction, amino acids, response surface method, chemometrics

## Abstract

The aim of this study is to establish a rapid and convenient microwave-assisted digestion method for sample pretreatment to evaluate amino acids in *Chebulae fructus* (CF). The microwave digestion method was optimized to extract amino acids from CF, and the differences in amino acids in CF from different origins and different processing states were analyzed and evaluated. The influences of digestion temperature, digestion time, and liquid–material ratio on extraction effect were investigated by sing factor test and response surface method (RSM), and the extraction conditions were optimized. The contents of 17 amino acids were determined by an automatic amino acid analyzer. The optimal digestion conditions were a digestion temperature of 150 °C, a digestion time of 18 min, and a liquid–material ratio of 65:1 (mL:g). Under these conditions, the total amino acid content of CF could reach 19.72 mg/g. CF from Lincang city of Yunnan province and unprocessed CF were considered to have higher nutritional value. The results of chemometric analysis showed that there were significant differences in the amino acid content in CF between Guangxi province, Dehong prefecture of Yunnan province, and Lincang city of Yunnan province, and six differential amino acids between the three origins were screened out. This study can provide references for the quality evaluation of the producing area, the extraction, and content research of amino acids of CF.

## 1. Introduction

*Chebulae fructus* (CF) is the dried ripe fruit of *Terminalia chebula Retz.* or *Terminalia chebula Retz.var.tomentella Kurt.*, which is mainly produced in India, Malaysia, Myanmar, and other countries. As noted in Encountering the Sources of the Classic of Materia Medica, “Raw CF clears the lungs and relieves cough, while roasted CF consolidates the spleen and stops diarrhea. Its bitter taste resolves phlegm and saliva, and its astringent nature prevents leakage”. It is distributed in Yunnan, Tibet, Guangdong, Guangxi and other places in China, and is known as the “king of herbal” [1]. CF has the effects of improving gastrointestinal function and relieving sore throat [2]. Modern research has found that CF contains phenolic acids, tannins, triterpenes, flavonoids, amino acids, and other chemical components [3,4]. Researchers established the quality evaluation method of CF based on its chemical composition, mainly focusing on phenolic acids and tannins, but less on its amino acids. However, the investigation of amino acids in CF is highly essential. By analyzing the content of amino acids in CF, we can enrich our understanding of its chemical composition, thereby providing a more comprehensive indicator for the quality evaluation of CF and further accurately assessing its quality and efficacy. CF possesses a variety of pharmacological activities, such as antibacterial, vasoconstrictive, antioxidant, and anti-tumor effects [5]. Although phenolic acids, tannins, and other secondary metabolites are considered the primary active constituents, amino acids may also exert certain pharmacological effects during the therapeutic process. For instance, amino acids can activate the NRF2 signaling pathway to exert antioxidant effects [6]; they can also play a role in anti-tumor activity by modulating the immune system or participating in cell signaling pathways [7]. More importantly, amino acids, as important nutrients for the body, play a vital role in the internal reaction of the body [8,9]. There are 20 kinds of amino acids involved in protein synthesis in the human body, which are divided into essential amino acids (EAA) and non-essential amino acids (NEAA), among which essential amino acids include Lysine (Lys), Tryptophan (Trp), Phenylalanine (Phe), Methionine (Met), Threonine (Thr), Isoleucine (Ile), Leucine (Leu) and Valine (Val). EAA cannot be synthesized by the body itself and must be ingested through diet [10]. Each amino acid has a corresponding pharmacological action. For example, Val can improve mitochondrial function and prevent oxidative stress [11]. Glu and Asp participate in glucose metabolism [12] and Arg can protect intestine and improve intestinal diseases [13]. Leu can regulate lipid metabolism and promote the body to utilize lipids [14]. Met is involved in cell signal transduction and related to neurodegenerative diseases [15,16].

The microwave digestion method involves producing heat energy through mutual friction between the sample molecules in the microwave electromagnetic field so as to accelerate the sample digestion [17,18]. Comparing the microwave digestion method with the conventional digestion method, it was found that the amino acid extraction results of the two methods were almost the same, and it was proven that the microwave digestion method could improve hydrolysis efficiency, and its accuracy was identical to the conventional hydrolysis method [19]. The microwave digestion method has the advantages of short processing time, low cost, and small sample consumption. There are many studies on the microwave digestion method for sample pretreatment [20,21,22].

At present, there are many methods to determine the content of amino acids, including automatic amino acid analyzer [23], high performance liquid chromatography (HPLC) [24], ultraviolet spectrophotometry (UV) [25], mass spectrometry (MS) [26], capillary electrophoresis (CE) [27], and so on. The sample pretreatment of HPLC is cumbersome and some derivatives are unstable. The reproducibility of UV is poor [28], and MS cannot distinguish L-leucine and L-isoleucine [29]. Automatic amino acid analyzer is the most commonly used method in laboratory, and its determination results are also considered to be “gold standard” [30]. The automatic amino acid analyzer method involves adsorbing different amino acids onto a cation exchange column and, in turn, using buffer solutions with different pH values to elute the amino acids. Their varying affinities for the resin result in different retention times. After separation, amino acids react with a derivatizing reagent (e.g., ninhydrin) to form colored products. These products have absorption peaks at specific wavelengths (e.g., 570 nm and 440 nm), measured by a photometric detector for quantification. Each amino acid has a unique retention time, which allows identification and quantification by comparing with known standards [31]. The method has a high degree of automation, simple operation, high reproducibility, and low operating cost.

Based on the microwave digestion and automatic amino acid analyzer methods, the extraction conditions of amino acids in CF were optimized, and the amino acid content from different origins and processing states was detected. Chemometrics analysis was carried out to compare the differences in amino acid composition among different CF, which provided some reference for the study of origin division, processing technology, quality evaluation, extraction, and evaluation of amino acids.

## 2. Materials and Methods

### 2.1. Instruments and Reagents

#### 2.1.1. Instruments

LA8080 automatic amino acid analyzer was purchased from Hitachi High-tech Science Co. (Tokyo, Japan). WX-6000 microwave digestion instrument was purchased from Shanghai Yiyao Technology Development Co. (Shanghai, China). RRH-A500 high-speed multifunctional grinder was obtained from Shanghai Yuanwo Industry & Trade Co. (Shanghai, China). 321LS Electronic Balance was acquired from Tianmei Yituo Laboratory Equipment Co. (Shanghai, China). The Milli-Q pure water meter was acquired from Millipore Company (Millipore, Milford, MA, USA), and the vacuum centrifuge concentrator was procured from Chongqing Lizhen Technology Co. (Chongqing, China).

#### 2.1.2. Reagents

The amino acid mixed standard solution (H-type) and ninhydrin chromogenic solution package were purchased from FUJIFILM Wako Pure Chemical Co. (Osaka, Japan). The amino acid mixed standard solution H-type included aspartic (Asp), threonine (Thr), serine (Ser), glutamic (Glu), proline (Pro), glycine (Gly), alanine (Ala), cystine (Cys), valine (Val), methionine (Met), isoleucine (Ile), leucine (Leu), tyrosine (Tyr), phenylalanine (Phe), lysine (Lys), histidine (His) and arginine (Arg). MIC BUFFER PH-KIT buffer system was procured from Japan Mitsubishi Chemical Co. (Tokyo, Japan). Hydrochloric acid was purchased from Chongqing Chuandong Chemical Group Co. (Chongqing, China).

#### 2.1.3. Sample Preparation

All CF samples were purchased from local medicinal materials markets and retail pharmacies in Guangxi Province, Dehong prefecture of Yunnan Province, and Lincang City of Yunnan Province, with a total of 23 batches of samples. All of them were identified as the mature fruit of *Terminalia Chebula Retz.* by Wang Jian, associate professor at the College of Traditional Chinese Medicine, Chongqing Medical University. The authentication was based on his experience and the 2020 edition of the Chinese Pharmacopoeia, through the examination of the shape, surface characteristics, texture, cross-sectional features, odor, cell morphology, and tissue structure. The samples were stored at room temperature in the laboratory of the College of Pharmacy, Chongqing Medical University. The specific sample information was shown in Table 1.

The processed CF is prepared using roasting. The specific steps are as follows: Clean the CF, wrap it with 3 to 4 layers of flour dough using the pill-making method, dry it until it is semi-dry, then place it in hot sand for roasting. When the flour coating turns dark yellow, remove it from the sand, peel off the flour coating, and crack open the CF to remove the kernel. The unprocessed CF is prepared by cleaning, pitting, and drying.

### 2.2. Solution Preparation

#### 2.2.1. Standard Solution

An appropriate amount of amino acid mixed standard solution with 0.0025 mM of each amino acid was accurately measured, diluted with 0.01 M hydrochloric acid to obtain 0.02, 0.04, 0.06, 0.08, 0.15, and 0.2 μM amino acid series standard solutions, and stored at 4 °C for later use.

#### 2.2.2. Sample Solution

An appropriate amount of CF was made into powder and passed through an 80-mesh sieve. A total of 0.2 g of CF powder was accurately weighed and placed in a PTFE digestion tube and 13 mL of 6 mol/L hydrochloric acid solution was added. The digestion tube was placed in the microwave digestion instrument and digested with the parameters of Table 2. After the digestion was completed, it was cooled to room temperature and taken out. The digested sample solution was transferred to a 50 mL volumetric flask and rinsed with ultrapure water 3 times. The washing solution was added to the volumetric flask, then made up to volume. The solution was filtered by 0.22 μm filter membrane, and 600 μL of the continuous filtrate was taken in a 1.5 mL EP tube, vacuum-dried at 60 °C for 6 h, and 600 μL of sodium citrate buffer solution (pH 2.2) was added and mixed. It was stored in refrigerator.

### 2.3. Chromatographic Condition

The amino acid content was detected by Hitachi LA8080 automatic amino acid analyzer. The analytical column was Hitachi Ion Exchange Resin #2622PH (4.6 mm × 60 mm, 3 μm) and the ammonia filter column was Hitachi Strongly acidic cation exchange resin column #2650 L (4.6 mm × 40 mm). The column temperature was 57 °C, and the temperature of post-column derivatization chamber was 135 °C. The injection volume was 10 μL. The detection wavelength of Pro was 440 nm, and the detection wavelength of other amino acids was 570 nm. The flow rate of mobile phase before column was 0.4 mL/min, including B1 (PH-1), B2 (PH-2), B3 (PH-3), B4 (PH-4), B5 (water) and B6 (PH-RG). The flow rate of post-column derivatization reagent was 0.35 mL/min, including R1 (ninhydrin reagent), R2 (buffer solution for ninhydrin reagent), and R3 (5% ethanol). The chromatographic workstation was Agilent OpenLab CDS (Agilent Technologies, Inc., Santa Clara, CA, USA). The gradient of flow and post-column derivatization reagent is shown in Table 3.

### 2.4. Comparison of Amino Acid Extraction Methods

#### 2.4.1. Microwave Digestion

The sample solution was prepared according to the content under Section 2.2.2, and the amino acid content was determined according to the chromatographic conditions under Section 2.3. Three parallel experiments were conducted.

#### 2.4.2. Conventional Digestion

The conventional digestion method was carried out according to the method specified in the national food safety standard [17] A total of 0.2 g of CF powder was accurately weighed in the hydrolysis tube and 10 mL of 6 mol/L hydrochloric acid solution was added. The hydrolysis tube was placed in an electrothermal blast incubator at 110 °C for 22 h and then removed and cooled to room temperature. The solution in the hydrolysis tube was transferred to a 50 mL volumetric flask, made up to volume, and filtered through a 0.22 μm filter membrane. Three parallel experiments were conducted. A total of 600 μL of the filtrate was accurately measured in a 1.5 mL EP tube, vacuum-dried at 60 °C for 6 h, and 600 μL of sodium citrate buffer solution (pH 2.2) was added to mix well. The amino acid content was determined according to the chromatographic conditions under “Section 2.3”.

#### 2.4.3. Water Bath Reflux Extraction

Water bath reflux extraction method was based on the method specified in determination of free amino acids in plants [32]. A total of 0.2 g of CF powder was accurately weighed in a 50 mL conical flask, 13 mL of water was added, heated in a 95 °C water bath for 10 min, and then taken out and cooled to room temperature. The subsequent amino acid extraction steps were consistent with those in Section 2.4.2.

#### 2.4.4. Ultrasonic Extraction

Ultrasonic extraction method referred to Cen Zhongyong’s method [33]. A total of 0.2 g of CF powder was accurately weighed in a 50 mL conical flask and then 13 mL of 25% ethanol solution was added. The ultrasonic temperature was 45 °C, and the ultrasonic time was 54 min. The conical flask was taken out and cooled to room temperature. The subsequent amino acid extraction steps were consistent with those in Section 2.4.2.

### 2.5. Single Factor Test

The effects of digestion temperature, digestion time, and liquid-solid ratio on the amino acid content of CF were investigated. Based on the method of Wang [17] and Ren Xiaoyu [34], the digestion temperature was 145 °C, the digestion time was 18 min, and the liquid–material ratio was 50:1 (mL:g). Two parameters were fixed and one of them was changed to study its effect on the total amino acid content of CF.

#### 2.5.1. Digestion Temperature Investigation

According to the content under “Section 2.2.2”, six sample solutions were prepared. 10 mL of 6 mol/L hydrochloric acid was added and digested at 110, 120, 130, 140, 150, 160 °C for 18 min, respectively. The amino acid content was determined according to the chromatographic conditions of “Section 2.3”.

#### 2.5.2. Digestion Time Investigation

Based on the content of “Section 2.2.2”, six sample solutions were prepared. 10 mL of 6 mol/L hydrochloric acid was added and digested at 145 °C for 5, 10, 15, 20, 25, and 30 min, respectively. The amino acid content was determined according to the chromatographic conditions of “Section 2.3”.

#### 2.5.3. Liquid–Material Ratio Investigation

According to the content under “Section 2.2.2”, six sample solutions were prepared, and 6 mol/L hydrochloric acid was added in the liquid–material ratio of 35:1, 45:1, 55:1, 65:1, 75:1, 85:1 (mL:g), separately, and digested at 145 °C for 18 min. The amino acid content was determined according to the chromatographic conditions of “Section 2.3” Table 2.

### 2.6. Response Surface Optimization Experiment

The RSM can be applied to evaluate the influence of a certain factor with the lowest number of experiments when multiple influencing factors coexist, and determine the most significant factors affecting the results [35,36]. The commonly used experimental design of RSM includes Box–Behnken experimental design, central composite design, and full factor design. The full factorial design has many experiments, a heavy workload, and is time-consuming. The number of central composite design experiments is more than that of Box–Behnken experimental design (except for 5 factors). The Box–Behnken experimental design only requires three levels of change factors, fewer experiments, and lower experimental costs [37,38].

Therefore, the Box–Behnken experimental design was adopted in this study. Based on the results of single factor test, Box–Behnken experimental design was carried out with digestion temperature (A), digestion time (B), and liquid-to-material ratio (C) as independent variables, and the amount of total amino acids extracted as response value (Table 4 and Table 5).

### 2.7. Method Validation

#### 2.7.1. Linearity, LODs, LOQs Experiment

The amino acid standard solutions with concentrations of 0.02, 0.04, 0.06, 0.08, 0.15, and 0.2 μM were taken and determined according to the chromatographic conditions. The limits of detection (LODs) and limits of quantification (LOQs) of each amino acid were, respectively, calculated at signal-to-noise ratios of 3 and 10.

#### 2.7.2. Precision and Repeatability Experiment

To ensure the reliability and accuracy of the experimental results, we conducted tests for both instrument precision and method repeatability. For instrument precision: The mixed standard solution of amino acids was accurately measured, and the solution was determined according to the chromatographic conditions. Parallel operation was performed 6 times, the peak area of each amino acid was recorded, and the relative standard deviations (RSDs) was calculated. For method repeatability, the sample solution was prepared and injected according to the chromatographic conditions. Five groups were operated in parallel to calculate the peak area of each amino acid.

#### 2.7.3. Recovery Experiment

The 0.1 g sample powder of CF with known amino acid content was accurately weighed, and an appropriate amount of amino acid mixed standard solution was added. The sample solution was prepared and injected and determined according to the chromatographic conditions. Parallel operation was performed 6 times, the peak area of each amino acid was recorded, and RSDs were calculated.

#### 2.7.4. Stability Experiment

The sample solution was prepared and injected at 0, 3, 6, 12, 18, and 24 h according to the chromatographic conditions. The peak area of each amino acid was calculated, respectively.

### 2.8. Determination and Evaluation of Content of Amino Acids in CF

CF sample solutions of different origins and processing states were prepared and determined according to the chromatographic conditions. Metaboanalyst 6.0 was used for chemometric analysis of the determination results, and SPSS 27 software was used for independent sample *t*-test.

## 3. Results and Discussion

### 3.1. Comparison Results of Amino Acid Extraction Methods

**Figure 1:**
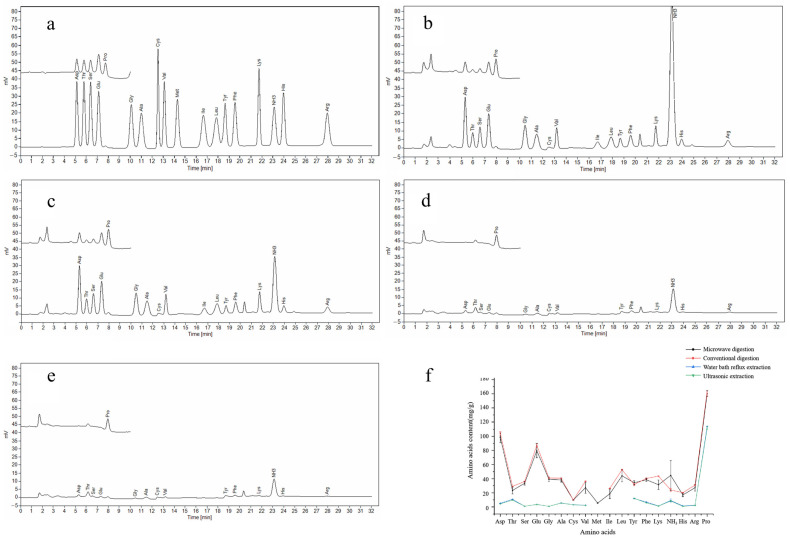
Chromatograms and amino acid content comparison of 4 extraction methods. (**a**) Chromatogram of the amino acids mixture standard solution. (**b**) Microwave digestion. (**c**) Conventional digestion. (**d**) Water bath reflux extraction. (**e**) Ultrasonic extraction. (**f**) amino acid content comparison of 4 extraction methods.

The extraction methods of amino acids include microwave digestion [39], conventional digestion [40], ultrasonic extraction [41], water bath reflux extraction [32,42], and so on. In order to select an optimal amino acid extraction method, this study selected four commonly used methods for comparison. The amino acid standard chromatogram is shown in Figure 1a. The type and quantity of amino acids are determined by analyzing the retention times and areas in the chromatogram. Figure 1b–e were the amino acid chromatograms detected by the four methods. 17 kinds of amino acids could be detected by microwave digestion. The conventional digestion method could detect 16 kinds of amino acids, and Met was not detected. Only 14 kinds of amino acids were detected by water bath reflux extraction and ultrasonic extraction, and Met, Ile, and Leu were not detected. The amino acid content measured by the four methods can be seen in Figure 1f, and the total amino acid content measured by the conventional digestion method was the highest, which was 19.83 ± 0.46 mg/g; the total amino acid content measured by microwave digestion was 18.10 ± 1.83 mg/g. Followed by water bath reflux extraction method, 4.25 ± 0.01 mg/g. The ultrasonic extraction method was the lowest, 4.09 ± 0.09 mg/g. Through the SPSS analysis results and Figure 1e, it was found that there was no significant difference in the amino acid content measured by the microwave digestion method and the conventional acid digestion method, while the amino acid content of water bath reflux extraction and ultrasonic extraction was only 1/4 of the microwave digestion. Therefore, considering the types of amino acids, total amino acid content, and time factors, microwave digestion was selected to extract amino acids from CF in this study.

### 3.2. Results of Single Factor Test

#### 3.2.1. Results of Digestion Temperature

**Figure 2:**
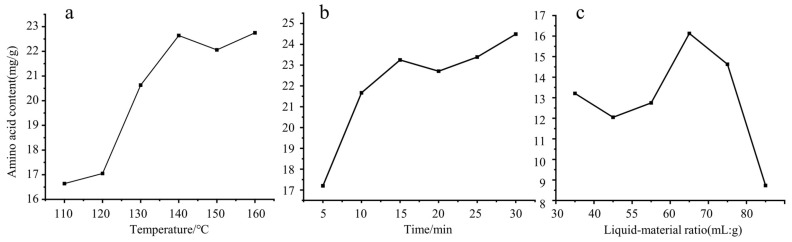
Effects of temperature (**a**), time (**b**), and liquid-material ratio (**c**) on amino acid content in CF.

The effect of digestion temperature on the content of total amino acids in CF was shown in Figure 2a. From the curve of Figure 2a, it was found that, from 110 °C to 140 °C, the total amount of amino acids in CF increased rapidly with the increase in digestion temperature. This may be because, when the temperature rises, the peptide bond of the protein breaks, producing more free amino acids [43]. When the digestion temperature was higher than 140 °C, the increase in temperature had no obvious effect on the increase in total amino acids. Compared with 140 °C, 150 °C had a slight decrease, while the amino acid content measured at 160 °C and 140 °C was almost the same. With the increase in temperature, some amino acids will be degraded and transformed, resulting in the decrease in amino acid contents [44]. Therefore, in order to protect the instrument and save time, response surface optimization experiments were conducted at 130 °C, 140 °C, and 150 °C.

#### 3.2.2. Results of Digestion Time

The effect of digestion time on the content of total amino acids in CF was shown in Figure 2b. The content of amino acids in CF increased with the increase in digestion time. However, when the time was more than 15 min, the content of amino acids in CF did not change significantly with the increase in digestion time. When the time was increased to 30 min, the total amount of amino acids in CF increased. Compared with 15 min, only 1 mg per gram was increased, but the time was doubled. With the increase in digestion time, the increase in amino acid content was not obvious. This phenomenon may be due to the increase in peptide bond cleavage caused by heating, which can increase the content of amino acids. However, due to the long heating time, amino acids undergo conversion; for example, Phe is converted into benzaldehyde, which leads to no obvious increase in amino acids [45]. So, 10, 15, and 20 min were chosen for response surface optimization experiment in this study.

#### 3.2.3. Results of Liquid-Material Ratio

The effect of the liquid–material ratio on the content of total amino acids in CF is shown in Figure 2c. As shown in Figure 2c, when the liquid–material ratio was between 35:1 (mL:g) and 65:1 (mL:g), the content of total amino acids in CF increased with the increase in liquidliquid–material ratio. When the liquid–material ratio was 65:1 (mL:g), the extraction amount of total amino acids in CF was the highest. When the liquid–material ratio continued to increase, the extraction amount of total amino acids in CF decreased. This may be because, with the increase in extraction solvent volume, the dissolution of other acid-soluble impurities increased, which affected the extraction of amino acids. Hence, the liquid–material ratio of 55:1 (mL:g), 65:1 (mL:g), and 75:1 (m L:g) were selected for response surface optimization experiment in this study.

### 3.3. Results of RSM Optimization Experiment

#### 3.3.1. Results of Box–Behnken Experimental Design

Based on the results of the single factor test, in order to further research the interaction between factors and obtain the best extraction process, a three-level and three-factor Box–Behnken experimental design was adopted to investigate the effects of digestion temperature (A), digestion time (B), and liquid–material ratio (C) on the extraction of total amino acids from CF. The three independent variables were maintained at three levels (−1, 0, +1) (Table 4) to design experiments, a total of 17 groups of experiments (Table 5). The sample solution was prepared and the content of amino acids was determined under the chromatographic conditions.

#### 3.3.2. Results of ANOVA and Significance Test

The total amino acids extraction amount of CF was taken as the response value, and the extraction process was optimized by RSM to find the best digestion temperature, digestion time and liquid–material ratio. The Box–Behnken experimental results were modeled and analyzed by Design-Expert 13 software. The ANOVA was used to ensure the significance of the model, and the lack of fit analysis was used to ensure the stability of the model. The values of *R*^2^ and *R*_adj_^2^ and their difference can prove the predictive ability of the model. In order to further explore the relationship between the independent variable and the response value, the regression model between the extraction amount of total amino acids in CF (y) and the three variables of digestion time (A), digestion time (B) and liquid–material ratio (C) was established: y = −168.72547 + 0.749227A + 3.07017B + 2.93008C − 0.0103342AB − 0.010487AC − 0.007617BC + 0.000862A^2^ − 0.030908B^2^ − 0.0094267C^2^. As shown in Table 6, the *p* value of model was less than 0.0001, which proved that the model was extremely significant. The *p* value of the lack of fit was 0.2938, which indicated that the difference in the model was not significant, and the unknown factors had little influence on it, so the model was stable. *R*^2^ was 0.9827, *R*_adj_^2^ was 0.9606, and the difference was less than 0.2, which meant that the model had a high degree of fitting, a small deviation from the actual value, and a certain predictive ability. From Table 6, it can be seen that digestion temperature (A), digestion time (B), liquid–material ratio (C), interaction between digestion temperature and liquid–material ratio (AC), quadratic term of digestion time (B2) and liquid–material ratio (C2) were extremely significant, indicating that these factors had a great influence on the extraction of total amino acids in CF. The order of the three factors affecting the extraction of total amino acids in CF was as follows: digestion temperature (A) > liquid–material ratio (C) > digestion time (B). In summary, the model could be used to reflect the response surface relationship between the total amino acid extraction amount and the digestion temperature, digestion time, and liquid–material ratio.

#### 3.3.3. Results of Response Surface Interaction Analysis

**Figure 3:**
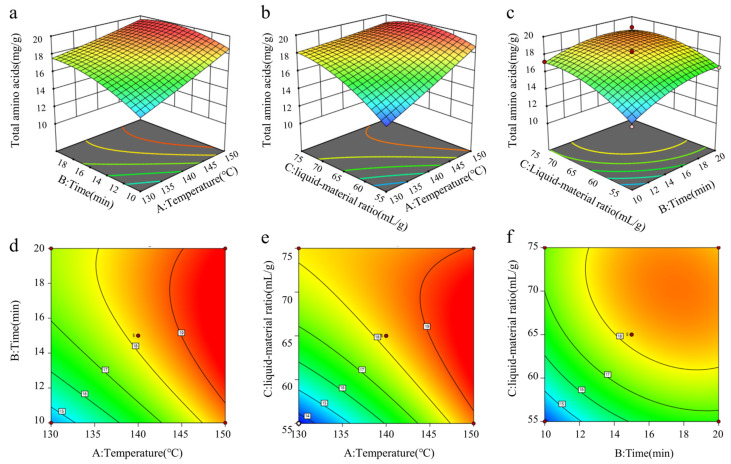
Effects of interaction of two factors on amino acid content in CF. Three-dimensional surface plots (**a**–**c**) and two-dimensional contour plots (**d**–**f**) showing the effects of temperature and time (**a**), temperature and liquid–material ratio (**b**), and time and liquid–material ratio (**c**) on the extraction amount of total amino acids from CF.

The extraction amount of total amino acids from CF was used as the response value, and digestion temperature, digestion time, and liquid–material ratio were taken as variables. The contour graph and response surface graph were drawn by Design-Expert13 software, which can intuitively show the interaction results of each variable [46]. When the contour line is closer to the ellipse and the response surface is steeper, it can be seen that the interaction between the two factors is more significant. The contour graph of AC (Figure 3b) was the closest to the ellipse, and the response surface graph (Figure 3e) was the steepest, indicating that the interaction between A and C was the most significant. AB followed (Figure 3a,d). In the interaction diagram of BC, the contour graph (Figure 3f) was close to the circle, the response surface graph (Figure 3c) was the smallest, and the interaction between variables was not significant. These results were also consistent with the significance analysis results in Table 6.

#### 3.3.4. Optimization and Verification of Extraction Process

The extraction process of total amino acids in CF was optimized by Design-Expert13 software. The optimized process was as follows: digestion temperature was 150 °C, digestion time was 17.59 min, and liquid–material ratio was 63.62:1 (mL:g). In order to facilitate the actual operation, the process was adjusted. The digestion temperature was 150 °C, digestion time was 18 min, and liquid–material ratio was 65:1 (mL:g), which was the final extraction process. At this time, the theoretical extraction amount of total amino acids predicted by the model was 19.87 mg/g. In order to verify the reliability of the extraction process, the process was tested. The extraction amount of amino acid was 19.72 mg/g, which was only 0.7% lower than the predicted value, indicating that the model was reliable.

### 3.4. Method Validation

#### 3.4.1. Results of Linearity, LODs, LOQs

The linear equation of each amino acid was calculated with concentration (*x*) as abscissa and amino acid content (*y*) as ordinate. The linearity results are shown in Table 7. The *R*^2^ values of all amino acids were greater than 0.999, indicating that the determination of amino acids met the linear relationship. The LODs and LOQs of 17 amino acids are listed in Table 7. Among them, the lowest LODs of Ser was 70.61 ng/mL, and the highest LODs of Pro was 887.0 ng/mL, illustrating that the response of chromatography to each amino acid was quite different.

#### 3.4.2. Results of Precision and Repeatability

The results of instrument precision were shown in Table 7. The RSDs of the peak areas of 17 amino acids ranged from 0.64% to 2.20%, indicating that the precision of the instrument was good. For method repeatability, the RSDs range of the peak area of 17 amino acids were 1.83%~7.48%, indicating that the method had good repeatability.

#### 3.4.3. Results of Recovery

The recovery results of each amino acid were shown in Table 8. The recoveries of 17 amino acids were in the range of 93.94~114.1%, with the RSDs from 0.47% to 5.01%, indicating that the method had a high recovery rate and good accuracy.

#### 3.4.4. Results of Stability

The stability of the sample was investigated by multiple injection analysis within 24 h. The RSDs of the peak area of 17 amino acids were between 0.37% and 2.94%, indicating that the sample was stable within 24 h.

### 3.5. Amino Acid Composition Analysis and Nutritional Value Evaluation

#### 3.5.1. Amino Acid Composition Analysis and Nutritional Value Evaluation of CF from Different Origins

Due to different growth conditions such as temperature, humidity, soil environment, and light, the effective components of plants in different regions will be different. Xia Huimin [47] proved that there were differences in the effective components of CF from different origins by HPLC fingerprint technology and gray correlation method. At present, the research on the differential components of CF from different origins focuses on phenolic acids such as gallic acid, corilagin, CF acid, and curculic acid [47,48]. Differences in amino acid composition have not been reported.

**Figure 4:**
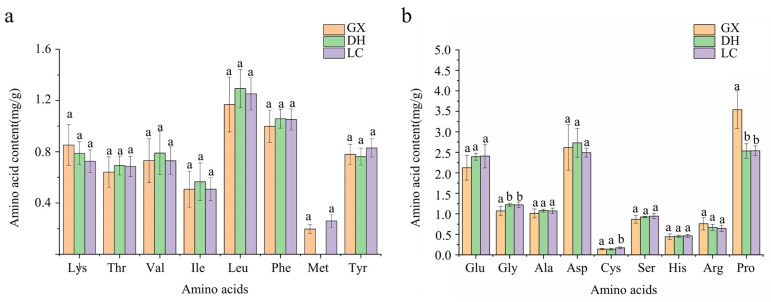
Results of amino acid content of CF from different areas. (**a**) Essential amino acid content. (**b**) Non-essential amino acid content. The different character of a,b means significance (*p* < 0.05); The same character of a, b means no significance (*p* > 0.05).

18 batches of CF samples were collected from GX, DH, and LC. The sample solution was prepared and the content of amino acids was determined according to the chromatographic conditions. The results were shown in Figure 4. Except for Met, which was not detected in GX-1, GX-7, DH-2, DH-3, DH-4, DH-5, 17 amino acids were detected in the other samples, including Met, Lys, Thr, Val, Ile, Leu, Phe, Met, Tyr, Glu, Gly, Ala, Asp, Cya, Ser, His, Arg, and Pro. The total amino acid content of GX was 18.40 ± 2.49 mg/g, and that of DH and LC was 18.13 ± 0.96 mg/g and 18.00 ± 1.27 mg/g, respectively. There was no significant difference in total amino acids content among the three producing origins, but there was a difference in single amino acid content. The average mass fractions of Lys, Arg, and Pro in GX were the highest. The average mass fractions of Thr, Val, Ile, Leu, Phe, Gly, Ala, and Asp in DH were the highest. The average mass fractions of Met, Tyr, Glu, Cys, Ser and His in LC were the highest. There were differences in the contents of Pro and Gly between GX and DH, Pro, Cys and Gly between GX and LC, and Cys between DH and LC. The ideal amino acid model published by FAO/WHO is that the ratio of essential amino acids (EAA) to total amino acids (TAA) is 35%~45%, and the ratio of EAA to non-essential amino acids (NEAA) is more than 60%. The greater the ratio, the higher the nutritional value [49]. The content of EAA in GX was 5.83 mg/g, the ratio of EAA/TAA was 31.58%, and the ratio of EAA/NEAA was 46.24%. The content of EAA in DH was 5.95 mg/g, EAA/TAA ratio and EAA/NEAA ratio were 32.76% and 48.88%, respectively. The EAA content of LC was 6.04 mg/g, the EAA/TAA ratio was 33.55%, and the EAA/NEAA ratio was 50.50%. The EAA/TAA and EAA/NEAA of the three origins were smaller than the ideal amino acid model, while the EAA/TAA and EAA/NEAA of LC were the highest, indicating that the nutritional value was relatively higher than the other two origins.

#### 3.5.2. Amino Acids Composition Analysis and Nutritional Value Evaluation of CF in Different Processing States

As one of the most commonly used Mongolian medicines, CF has low utilization rate of direct medicine, and its processed products are commonly used in clinic [50]. The processing methods of CF include madder, Stellera chamaejasme, simmering, stir-frying, boiling, wine steaming, sand blanching, and so on [51,52]. The heating, boiling, extrusion, baking and other steps in the processing will cause the content of amino acids to increase or decrease [53,54]. However, some TCM need to be processed before they are used. After processing, they can enhance or delay the efficacy, reduce toxicity, improve odor, and make the drugs easy to transport and store [55]. Studies have proven that there were differences in effective components and pharmacological effects of CF before and after processing. For example, the gallic acid content of CF increased by flour or wheat roasting [56]. The efficacy of CF processed at different temperatures was different, and the processing effect at 290 °C was the best [57].

**Figure 5:**
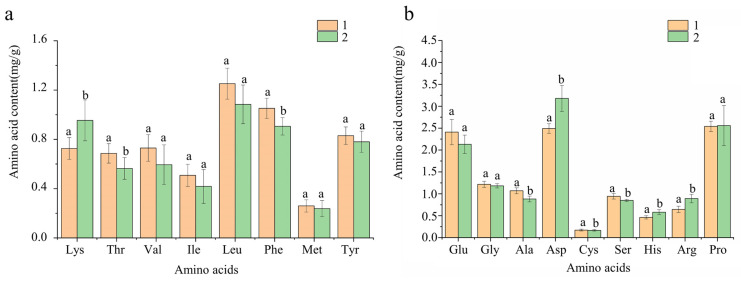
Results of amino acid content in of CF in different processing states. (**a**) Essential amino acid content. (**b**) Non-essential amino acid content. The different character of a,b means significance (*p* < 0.05); The same character of a, b means no significance (*p* > 0.05).

The results of amino acid content in Figure 5 showed that both processed and unprocessed CF contained 17 kinds of amino acids, including Met, Lys, Thr, Val, Ile, Leu, Phe, Met, Tyr, Glu, Gly, Ala, Asp, Cya, Ser, His, Arg and Pro. The TAA of processed CF was 17.96 ± 1.63 mg/g, and that of unprocessed CF was 18.01 ± 1.27 mg/g. Through SPSS software analysis, the results showed that there was no difference in the TAA of CF before and after processing. However, there were differences in the content of single amino acid between them. The average contents of Asp, Lys, Arg, Pro and His in the processed products were the highest, while the average contents of Thr, Ser, Glu, Gly, Ala, Cys, Val, Met, Ile, Leu, Tyr and Phe in the unprocessed products were the highest. According to the results of SPSS analysis, Asp, Thr, Ala, Arg, His, Lys, Phe, Ser and Cys were the differential amino acids of CF before and after processing (*p* < 0.05). The EAA in processed products was 5.54 mg/g, the EAA/TAA ratio was 30.75%, and EAA/NEAA ratio was 44.52%. The EAA in unprocessed products was 6.04 mg/g, the ratio of EAA/TAA and EAA/NEAA were 33.55% and 50.50%, which was closer to the standard published by FAO/WHO. Therefore, this study believed that the nutritional value of unprocessed CF was higher. The content of EAA in the processed CF was low, which may be due to the high temperature of sand blanching and the long time of stir-frying, which led to the reaction and degradation of some amino acids and other substances.

### 3.6. Chemometric Analysis

#### 3.6.1. Amino Acid Chemometric Analysis of CF from Different Origins

In order to explore the relationship between different origins and amino acid content in CF, the data in Figure 4 were analyzed by chemometric analysis with MetaboAnalyst 6.0.

**Figure 6:**
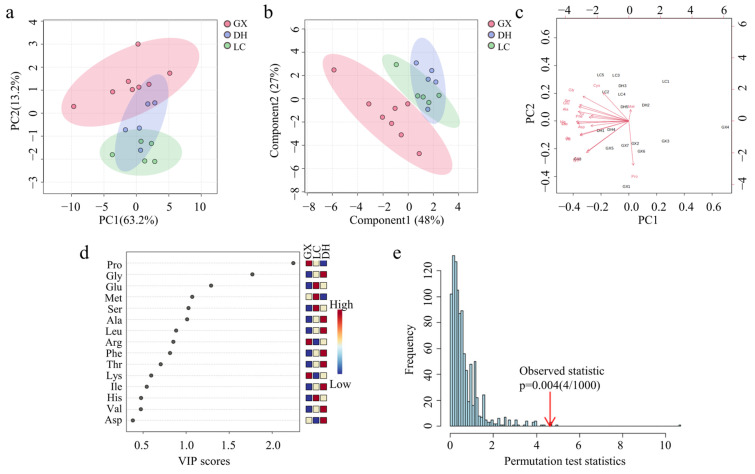
Results of chemometrics analysis of different origins. (**a**) PCA score plot. (**b**) PLS-DA score plot. (**c**) Biplot. (**d**) VIP plot. (**e**) Permutation scatter plot.

The variance contribution rates of principal component 1 (PC1) and principal component 2 (PC2) were 63.2% and 13.2%, respectively, with a cumulative rate of 76.4%, which indicated that PC1 and PC2 could explain most of the information of the original variables. So, the content of amino acids in CF from different origins could be compared and analyzed by using the first two principal components. The results of principal component analysis (PCA) from three origins showed that samples from the same province could be well clustered together, and samples from different provinces could be well distinguished (Figure 6a). There was partial overlap between DH and LC. The reason may be that the samples of the two groups were from Yunnan Province, and there were some similarities, so they could not be completely distinguished. It could be considered that the difference in amino acid content in CF samples was related to the geographical environment of growth.

To further study the differences between CF from different origins, partial least squares discriminant analysis (PLS-DA) was used for analysis. PLS-DA score plot (Figure 6b) showed that the model could distinguish the samples from Guangxi province and Yunnan province well. The samples from Lincang and Dehong in Yunnan partially overlapped, which was consistent with PCA, proving that there were obvious differences in amino acid types and contents of CF in different provinces. There were certain similarities in the same province due to the similarity in growth environment. Each point in the Biplot (Figure 6c) represents a sample, and the distance between sample points can indicate the similarity between samples. GX was concentrated below the graph, while DH and LC were above it, which also proved that there were differences in amino acid compositions and contents of CF from different origins. The Variable Importance in Projection (VIP) was analyzed (Figure 6d). The bigger the VIP value of an amino acid, the greater its influence on the content difference between different origins [58]. With VIP value greater than 1 as the index, six differential amino acids can be screened out, namely Pro, Gly, Glu, Met, Ser, and Ala, which could be used to distinguish CF from different origins. Over-fitting was likely to occur when PLS-DA was used for analysis, so the model was tested 1000 times (Figure 6e), and the results showed that the model had not been over-fitted.

#### 3.6.2. Amino Acid Chemometric Analysis of CF in Different Processing States

**Figure 7:**
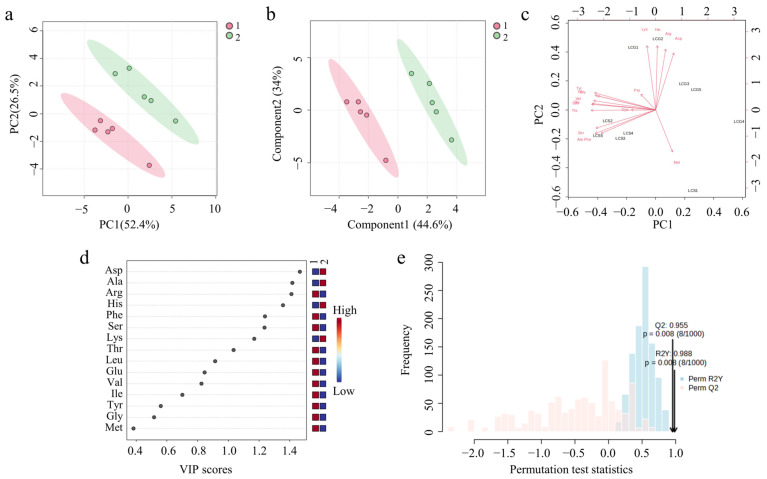
Results of chemometrics analysis of different processing states. (**a**) PCA score plot. (**b**) PLS-DA score plot. (**c**) Biplot. (**d**) VIP plot. (**e**) Permutation scatter plot.

MetaboAnalyst 6.0 was used for PCA of CF in different processing states. The variance contribution rates of PC1 and PC2 were 52.4% and 25%, respectively, and the cumulative contribution rate was 78.9%. It was indicated that PC1 and PC2 could explain most of the information of the original variables, and the first two principal components could be used to compare and analyze the amino acid content of CF in different processing states. PCA score plot (Figure 7a) distinguished the samples well, and divided them into two groups according to the results. Group 1 was unprocessed CF and Group 2 was processed CF. There were obvious differences between the two groups, and it could be considered that the difference in amino acid content in CF samples was closely related to the processing technology.

The PLS-DA was used to further analyze the differences in processed CF and unprocessed CF. The PLS-DA score plot (Figure 7b) separated processed CF from unprocessed CF, which proved that there was a significant difference in the amino acid content between the two. In the Biplot (Figure 7c), the unprocessed and processed CF were distributed in two areas, which also confirmed the above conclusions. The VIP value of the model was analyzed (Figure 7d), and eight amino acids were selected with VIP greater than 1 as the index, including Asp, Arg, Ala, His, Phe, Ser, Lys, and Thr, which can be used to distinguish CF in different processing states. The model has been tested 1000 times (Figure 7e), Q^2^ = 0.955, R^2^Y = 0.988, and the difference was less than 0.2, which proved that the fitting effect was good and there was no over-fitting.

## 4. Conclusions

In this study, the effects of digestion temperature, digestion time, and liquid–material ratio on the extraction of amino acids from CF were investigated by single factor test, and the extraction conditions were optimized by response surface optimization experiment. The optimized digestion conditions were as follows: digestion temperature was 150 °C, digestion time was 18 min, and liquid–material ratio was 65:1 (mL:g). The optimized microwave extraction method was faster and simpler. The optimized process was used to extract and determine the amino acids in CF from different origins and processing states. The results showed that 17 amino acids could be detected in GX and LC, while most of DH could only detect 16 amino acids, and 17 amino acids could be detected in processed and unprocessed CF from LC. The nutritional value of different CFs were evaluated according to the standards published by FAO/WHO. The results showed that processed CF Plhad the best nutritional value among the three origins; the nutritional value of unprocessed CF was higher in different processing states. MetaboAnalyst6.0 was used for Chemometric analysis. It was found that the amino acids of CF from different origins and different processing states were indeed different. It was confirmed by VIP value greater than 1 that Pro, Gly, Glu, Met, Ser, and Ala could be used to distinguish CF from different origins, and Asp, Arg, Ala, His, Phe, Ser, Lys, and Thr could be used to distinguish CF from different processing states. The compositions and contents of amino acid in CF from different origins and processing states were different, which could be used to distinguish different producing origins of CF and evaluate the nutritional value.

## Figures and Tables

**Table 1:** Sample information.

NO.	Sample	Collection Region	Processed or Not
1	GX-1	Guangxi Province (GX)	unprocessed
2	GX-2	Guangxi Province (GX)	unprocessed
3	GX-3	Guangxi Province (GX)	unprocessed
4	GX-4	Guangxi Province (GX)	unprocessed
5	GX-5	Guangxi Province (GX)	unprocessed
6	GX-6	Guangxi Province (GX)	unprocessed
7	GX-7	Guangxi Province (GX)	unprocessed
8	GX-8	Guangxi Province (GX)	unprocessed
9	DH-1	Dehong prefecture, Yunnan province (DH)	unprocessed
10	DH-2	Dehong prefecture, Yunnan province (DH)	unprocessed
11	DH-3	Dehong prefecture, Yunnan province (DH)	unprocessed
12	DH-4	Dehong prefecture, Yunnan province (DH)	unprocessed
13	DH-5	Dehong prefecture, Yunnan province (DH)	unprocessed
14	LC-1	Lincang City, Yunnan province (LC)	unprocessed
15	LC-2	Lincang City, Yunnan province (LC)	unprocessed
16	LC-3	Lincang City, Yunnan province (LC)	unprocessed
17	LC-4	Lincang City, Yunnan province (LC)	unprocessed
18	LC-5	Lincang City, Yunnan province (LC)	unprocessed
19	LC-6	Lincang City, Yunnan province (LC)	processed
20	LC-7	Lincang City, Yunnan province (LC)	processed
21	LC-8	Lincang City, Yunnan province (LC)	processed
22	LC-9	Lincang City, Yunnan province (LC)	processed
23	LC-10	Lincang City, Yunnan province (LC)	processed

**Table 2:** Parameters of microwave digestion instrument.

Step	Temperature/°C	Time/min	Pressure/atm
1	80	3	10
2	120	3	20
3	150	18	30

**Table 3:** Flow and post-column derivatization reagent gradient table.

Time (min)	%B1	%B2	%B3	%B4	%B5	%B6	%R1	%R2	%R3	Pump 2 Flow Rate (mL/min)
0.0	100	0	0	0	0	0	50	50	0	0.35
2.5	100	0	0	0	0	0	50	50	0	
2.6	0	100	0	0	0	0	50	50	0	
4.5	0	100	0	0	0	0	50	50	0	
4.6	0	0	100	0	0	0	50	50	0	
12.8	0	0	100	0	0	0	50	50	0	
12.9	0	0	0	100	0	0	50	50	0	
27.0	0	0	0	100	0	0	50	50	0	
27.1	0	0	0	0	0	100	50	50	0	
32.0	0	0	0	0	0	100	50	50	0	
32.1	0	0	0	0	0	100	0	0	100	
33.0	0	0	0	0	0	100	0	0	100	
33.1	0	100	0	0	0	0	0	0	100	
34.0	0	100	0	0	0	0	0	0	100	
34.1	100	0	0	0	0	0	0	0	100	
37.0	100	0	0	0	0	0	0	0	100	
37.1	100	0	0	0	0	0	50	50	0	
53.0	100	0	0	0	0	0	50	50	0	

**Table 4:** Box–Behnken experimental design levels and factors table.

Level	Factors		
	A: Temperature/℃	B: Time/min	C: Liquid–Material Ratio (mL:g)
−1	130	10	55:1
0	140	15	65:1
+1	150	20	75:1

**Table 5:** Design and results of Box–Behnken experimental design.

NO.	Factors			Amino Acid Content mg/g
	A/°C	B/min	C/mL:g	
1	130	10	65:1	14.56
2	150	10	65:1	18.73
3	130	20	65:1	17.36
4	150	20	65:1	19.46
5	130	15	55:1	13.80
6	150	15	55:1	18.92
7	130	15	65:1	17.90
8	150	15	75:1	18.82
9	140	10	55:1	13.55
10	140	20	55:1	16.57
11	140	10	75:1	17.20
12	140	20	75:1	18.69
13	140	15	65:1	18.37
14	140	15	65:1	18.31
15	140	15	65:1	17.66
16	140	15	65:1	18.36
17	140	15	65:1	18.38

**Table 6:** Results of ANOVA and significance of response surface optimization model.

Source	Sum of Squares	df	Mean Square	F-Value	*p*-Value	Significance
Model	51.57	9	5.73	44.31	<0.0001	**
A-Temperature	18.92	1	18.92	146.31	<0.0001	**
B-Time	8.08	1	8.08	62.47	<0.0001	**
C-liquid–material ratio	11.93	1	11.93	92.21	<0.0001	**
AB	1.07	1	1.07	8.26	0.0239	*
AC	4.4	1	4.4	34.02	0.0006	**
BC	0.5803	1	0.5803	4.49	0.0719	-
A^2^	0.0313	1	0.0313	0.2417	0.638	-
B^2^	2.51	1	2.51	19.44	0.0031	**
C^2^	3.74	1	3.74	28.93	0.001	**
Residual	0.9053	7	0.1293			
Lack of Fit	0.5148	3	0.1716	1.76	0.2938	-
Pure Error	0.3905	4	0.0976			
Cor Total	52.48	16				
*R* ^2^	0.9827		*R*_adj_^2^	0.9606		

Note: - means not significant (*p* > 0.05); * means significant (*p* < 0.05); ** means very significant (*p* < 0.01).

**Table 7:** Results of linearity, LODs, LOQs and precision.

NO.	Amino Acid	Linear Equation	*R* ^2^	LODs ng/mL	LOQs ng/mL	Precision RSD (%)
1	Asp	*y* = 1343.7*x* − 0.2637	0.9999	86.94	26.08	0.47
2	Thr	*y* = 1200.9*x* − 0.0466	0.9999	80.00	24.00	0.34
3	Ser	*y* = 1075.8*x* − 0.9075	0.9999	70.61	21.18	0.32
4	Glu	*y* = 1476.7*x* + 0.3726	0.9999	112.8	33.83	0.34
5	Gly	*y* = 751.04*x* + 0.0118	0.9999	79.48	23.84	0.78
6	Ala	*y* = 868.63*x* + 1.5939	0.9999	122.6	36.77	1.07
7	Cys	*y* = 2483.2*x* − 2.673	0.9995	119.2	35.77	0.70
8	Val	*y* = 1179.8*x* − 0.4082	0.9999	84.10	25.23	0.73
9	Met	*y* = 1518.4*x* − 0.3548	0.9999	152.5	45.75	0.68
10	Ile	*y* = 1332.3*x* − 0.9097	0.9999	187.9	56.37	0.56
11	Leu	*y* = 1245.7*x* + 4.1616	0.9997	219.7	65.92	1.91
12	Tyr	*y* = 1880.2*x* − 0.8519	0.9998	213.6	64.08	1.70
13	Phe	*y* = 1720*x* − 4.2533	0.9999	171.5	51.44	0.58
14	Lys	*y* = 1516.2*x* − 2.3157	0.9998	84.75	25.42	0.45
15	His	*y* = 1585.9*x* − 1.2187	0.9999	140.1	42.03	1.44
16	Arg	*y* = 1779.4*x* − 2.8503	0.9999	251.8	75.53	0.54
17	Pro	*y* = 1188.4*x* − 2.3913	0.9999	887.0	266.10	0.40

**Table 8:** Results of recovery rate.

NO.	Amino Acids	Amount of the Standards/mg	Recovery Rate/%	RSD/%
1	Asp	0.28	114.1	4.53
2	Thr	0.30	96.26	2.86
3	Ser	0.26	100.3	2.79
4	Glu	0.37	100.3	4.17
5	Gly	0.19	96.60	3.33
6	Ala	0.22	97.40	2.78
7	Cys	0.60	99.90	0.47
8	Val	0.29	94.64	3.39
9	Met	0.37	93.94	0.70
10	Ile	0.33	95.00	2.48
11	Leu	0.33	95.64	4.04
12	Tyr	0.45	96.58	1.25
13	Phe	0.41	95.80	1.21
14	Lys	0.37	94.52	2.88
15	His	0.39	95.27	1.05
16	Arg	0.44	95.27	2.01
17	Pro	0.29	103.4	5.01

## Data Availability

The original contributions presented in the study are included in the article, further inquiries can be directed to the corresponding author.
